# Experimental insights into energy savings and future directions of drag reducing polymers in multiphase flow pipelines

**DOI:** 10.1038/s41598-023-37543-w

**Published:** 2023-06-30

**Authors:** Ihab H. Alsurakji, Abdelsalam Al-Sarkhi, Amjad El-Qanni, Ayman Mukhaimar

**Affiliations:** 1 Department of Mechanical Engineering, An-Najah National University, Nablus 7, Palestine; 2 Mechanical Engineering Department and Center for Integrative Petroleum Research, Dhahran, Saudi Arabia; 3 Department of Chemical Engineering, An-Najah National University, Nablus 7, Palestine; 4 School of Computing Technologies, RMIT University, Melbourne, Australia

**Keywords:** Chemical engineering, Mechanical engineering

## Abstract

Frictional pressure drop has been grasping the attention of many industrial applications associated with multi-phase and academia. Alongside the United Nations, the 2030 Agenda for Sustainable Development calls for the exigency of giving attention to economic growth, a considerable reduction in power consumption is necessary to co-up with this vision and to adhere to energy-efficient practices. Thereinto, drag-reducing polymers (DRPs), which do not require additional infrastructure, are a much better option for increasing energy efficiency in a series of critical industrial applications. Therefore, this study evaluates the effects of two DRPs—polar water-soluble polyacrylamide (DRP-WS) and nonpolar oil-soluble polyisobutylene (DRP-OS)—on energy efficiency for single-phase water and oil flows, two-phase air–water and air-oil flows, and three-phase air–oil–water flow. The experiments were conducted using two different pipelines; horizontal polyvinyl chloride with an inner diameter of 22.5 mm and horizontal stainless steel with a 10.16 mm internal diameter. The energy-efficiency metrics are performed by investigating the head loss, percentage saving in energy consumption (both per unit pipe length), and throughput improvement percentage (%TI). The larger pipe diameter was used in experiments for both DRPs, and it was discovered that despite the type of flow or variations in liquid and air flow rates, there was a reduction in head loss, an increase in energy savings, and an increase in the throughput improvement percentage. In particular, DRP-WS is found to be more promising as an energy saver and the consequent savings in the infrastructure cost. Hence, equivalent experiments of DRP-WS in two-phase air–water flow using a smaller pipe diameter show that the head loss drastically increases. However, the percentage saving in power consumption and throughput improvement percentage is significantly compared with that found in the larger pipe. Thus, this study found that DRPs can improve energy efficiency in various industrial applications, with DRP-WS being particularly promising as an energy saver. However, the effectiveness of these polymers may vary depending on the flow type and pipe diameter.

## Introduction

Recent years have witnessed a substantial increase in interest in the subject of drag-reducing polymers (DRPs) in multi-phase flow pipes as a result of this need for energy-efficient and energy-saving technologies^1^. These polymers have the potential to reduce power consumption and improve energy management in various single and multi-phase flow applications. The flow pattern, or the arrangement of the different phases within the pipeline, can significantly impact the effectiveness of these polymers. Understanding how DRPs behave in different flow patterns and how they may help to advance energy-saving strategies, particularly at the industrial level, is crucial in this context. Consequently, it is imperative to have a thorough grasp of single- and multi-phase flows in pipes in order to improve energy efficiency in crucial industrial applications. Such applications include the nuclear industry, high-temperature heat exchangers, chemical reactors, oil and gas transportation, sustenance transformation, mining, pharmaceuticals, transportation of pulverized coal in fuel pipes, etc.^2^. Herein, the standard requirement is that these pipes transport a given fluid with a smaller frictional pressure drop (drag) and hence save energy. The use of DRPs, which are also called drag-reducing agents DRAs, instead of applying several pumps and/or loops, is a much better option to increase the efficiency as well as the throughput.

Intensive studies on drag reduction in single and multi-phase flows reveal that DRPs reduce the pressure drop and change the spatial distribution of fluids (flow pattern) in the pipe and at the boundaries. According to Choi and Jhon^3^, the most effective DRPs have flexible/noncrystalline linear structures and ultrahigh high molecular weights, usually greater than Mg/mol. Several review reports consolidate the huge number of DRA-related single-phase^4^ and multi-phase flow^5–7^ publications. A moderate summary is provided below.

The pioneering work in a single-phase flow is the study by Toms^8^, which implies that a tiny amount of DRP has a substantial effect on any solvent in reducing the friction pressure drop in a turbulent flow. Selected subsequent studies include contributions by the type of drag-reducing agents (DRAs). These DRAs include DRPs^9–12^, surfactants^13–16^, fibers^17^, and microbubbles^18^. Most of them considered the effect of sudden injection of DRAs, either by homogenous or heterogenous method, while others attempted to find a correlation expression for the percentage drag reduction (%DR) achieved. However, among these agents, DRPs have been considered the most efficient^19^. Additionally, a literature survey of the published work on DRPs in multi-phase flow pipelines assured the growing interest^19–25^. In addition to drag reduction, the aforementioned studies reported that DRPs can change the configurations of the co-existing phases. For instance, Tan et al.^20^ investigated the effect of adding polyacrylamide, with a concentration of 100 ppm and a molecular weight of 19 Mg/mole, on different flow patterns of oil–water two-phase flows. This investigation used a horizontal acrylic pipe with an inner diameter of 25.4 mm. The flow patterns examined were stratified, stratified with mixing at the interface, oil and dispersion of oil in water, dispersion of water in oil, dispersion of oil in water and water, and dispersion of oil in water. The superficial velocities of oil and water ranged from 0.2–1.5 m/s and 0.1–1.6 m/s, respectively. They found that the DRP could extend the transition boundaries of all the flow patterns except for the dispersion of water in the oil flow pattern. Ayegba et al.^24,25^ studied the influence of hydrolyzed polyacrylamide on different flow patterns of oil–water two-phase flow. These flow patterns include stratified, stratified wavy, dual continuous, bubbly, dispersed oil in water and water, dispersed oil and water, and dispersed water in oil. The straight portion of the test section has an internal diameter of 19 mm and is made of transparent polyvinyl chloride material. The superficial velocities of oil and water ranged from 0.04–0.95 m/s and 0.13–1.10 m/s, respectively. They found that adding DRP forced partial and/or complete flow stratification.

Furthermore, the mechanism of DRPs and the level of drag reduction have been investigated extensively and attributed to the flowrate^26^, the concentration of DRPs^27^, temperature^28,29^, flow channel geometry^30,31^, flow orientation^32^, phase distribution^20,33^, viscoelasticity^34^, and DRP molecular weight and its bond structure^19,35^. According to Karami and Mowla^35^, two opinions explain the existing drag reduction mechanisms. One of them is explained by^36^, which is based on the increment of the thickness of the viscous sub-layer caused by the protraction of coiled polymer molecules. The other is proposed by^37,38^, who ascribe the drag reduction to the elastic properties of DRPs. Recent studies by Alsurakji, et al.^39,40^ indicated that the performance of DRPs depends strongly on their hydrodynamic size, chemical structure, concentration, fluid flow rate, fluid flow pattern, and turbulence intensity. Particularly, the nonpolar DRP-OS with relatively straight chains was found to be more susceptible to shear degradation than the polar DRP-WS copolymer with longer branches and ion pairs surrounding its backbone. Their capacity to modify flow patterns, lessen drag, and lower pressure gradients in distinct fluid flow phases was significantly impacted by these variations in their structural properties. Additionally, it was discovered that the copolymer's concentration and the rates of liquid and air movement had an impact on how well it performed. Notably, depending on the precise experimental circumstances, the airflow rate could improve or worsen the performance of the copolymer. These findings were manifested in subsequent investigations by using particle image velocimetry (PIV) technique^41^.

Evidently, the presence of DRPs in pipelines attracted much interest and is explicitly investigated. Still, a limited number of published research in the open literature highlighted the application of DRPs in the industry as a flow improver. Burger et al.^42^ studied the effect of adding DRP on crude oil transportation in the trans-Alaska pipeline system (TAPS), having a relatively large diameter of about 356 mm and 1220 mm. The main finding was the improvement in flow rate by adding 10-ppm polymer to 1300 km pipelines. Gyr and Bewersdorff^43^ reported that the DRPs could be used as a flow improver in pipelines. They classified flow improvement into two categories. The first category considers that the energy level remains the same and DRPs increase the flow. As a result, the throughput increases. The second one conceives that the flow rate remains the same and DRPs minimize the pumping energy. Therefore, they concluded that DRPs could help increase the system's capacity and save power. Karami and Mowla^44^ performed a partial energy analysis by investigating the effect of adding DRP in single-phase crude oil pipelines on the pressure drop and the head loss. They remarked that using DRP helps decrease the head loss of the flow. A recent study by Al-Wahaibi et al.^32^ investigated the existence of DRP in two-phase liquid–liquid flow, reported the energy analysis in terms of head loss, pumping power, and flow rate. A reduction in the pumping power was obtained, which is needed to conquer the head loss, hence, increasing the throughput.

The literature review mentioned above demonstrates that there is only a limited understanding of how DRPs can reduce energy consumption by utilizing energy analyses for single- and multi-phase flows with various types of DRPs and/or pipe sizes. The main reason for doing the present study was the lack of research in this crucial field of study. By conducting experiments under various operational settings that span single-phase, two-phase, and three-phase flow with and without DRPs, the goal is to do a thorough energy analysis. The following will be considered:

i. Two structurally different DRPs—one polar and the other nonpolar—with varying concentrations;
ii. Two different pipe diameters.
iii. Single-phase, two-phase, and three-phase flows, consisting of (as appropriate) air, oil, and water with and without DRPs and their corresponding flow patterns, slug, and annular flow, will be experimented. Their flow rates were varied as follows:
  1. 0.0030–0.0265 m^3^ min^−1^ for the water phase;
  2. 0.0010–0.0350 m^3^ min^−1^ for the oil flow phase; and
  3. 0.0599–0.488 m^3^ min^−1^ for air.

The energy analysis will cover head loss, percentage saving in power consumption (both per unit pipe length), and throughput improvement percentage (%TI). Fluids' properties like temperature, viscosity, and density were kept constant for this investigation.

## Experimental set-up and procedure

### Set-up

Two experimental facilities, capable of studying the influence of different pipe diameters and operational conditions on energy analysis, were used to achieve the objectives of this study.

**Figure 1:**
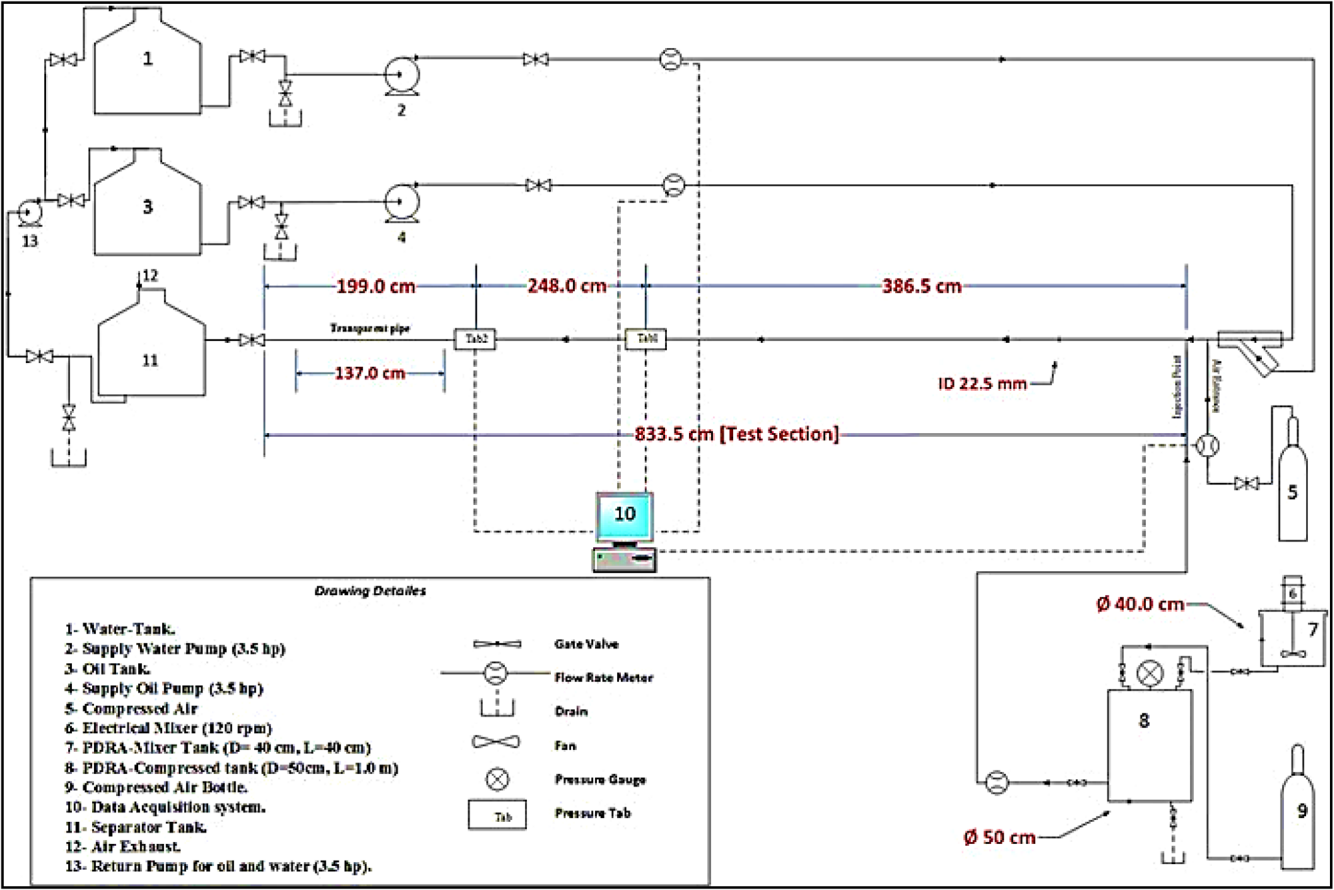
Schematics of the larger diameter multi-phase flow facility.

Figure 1 shows the larger diameter multi-phase flow facility. It consists of two tanks, each with a supply pump (that supplies oil and water), drag-reducing polymer tanks, separation tank, return pump, pipes including a transparent test section, an air compressor, flow meter sensors, pressure transmitter sensor, and data acquisition system. The output of the sensors (flow transmitter sensors and wet/wet differential pressure transmitter sensor) was acquired by recording the data at a rate of 1 Hz duration during the test period. A "LABVIEW" interface program was used to save the acquired data.

All experiments reported in this study were conducted using atmospheric air as a gas phase, tap water, and ESCAID^TM^110. ESCAID^TM^110—a particular type of kerosene—was applied as the oil phase because of its stability at the operating conditions of the loop and its low density that easily separates it from water. The properties of air, tap water, and oil are presented in Table 1.

**Table 1:** Properties of oil, tap water, air, DRP-WS, and DRP-OS.

Substance	Property	Typical value	Test based on
Oil (ESCAID™ 110)	Specific gravity @ 15.6/15.6 °C	0.790–0.810	ASTM D4052
	Kinematic viscosity @ 40.0 °C, cSt	1.50–1.75	ASTM D445
Tap water	Density @ 25 °C, kg/m^3^	998	NIST
	Viscosity @ 25 °C, Pa s	0.000985	NIST
Air	Density @ 25 °C, kg/m^3^	1.2	NIST
	Viscosity @ 25 °C, Pa s	0.000018	NIST

	Description		
DRP-WS	Product name Appearance Molecular weight Bulk density PH of 0.5% solution Solubility	Polyacrylamide (PAM) Off-white granular solid 8.0–10.0 Mg/mol 0.7 g/cm^3^ Approx. 3.5 Water-soluble	
DRP-OS	Product name Supplier Molecular weight Description Specific gravity Solubility	Polyisobutylene (PIB) Scientific Polymer Products, Inc 2.8 mg/mol Odorless clear slab 0.92 at 20 °C Oil-soluble	

DRP-WS is made of a copolymer of acrylamide and proprietary quaternized cationic monomer with the ability to be soluble in water. Following the method described by^40^, a 1000 ppm master solution of DRP-WS was prepared by dissolving it in tap water using a 50 L stainless steel tank, 120 rpm low-speed mixer, and a mixing period lasted for six h. In addition, a 1440 ppm master solution of DRP-OS which has an ultrahigh molecular weight (2.8 Mg/mol) and a linear rubbery (amorphous) structure^3^, was used as the oil-soluble DRP. This quantity was prepared by following same procedure used to prepare DRP-WS. Both DRPs were injected into the test section through a 2 mm side hole located 60 cm far from the mixing point of oil and water, and their effect on fluid flow were investigated by varying the feed concentration of the master solution and recording the corresponding pressure drops for the three cases, which are single-, two-, and three-phase flows. Table 1 summarizes the properties of the above DRPs.

The effect of DRP-WS and DRP-OS on fluid flow was investigated by varying the solution concentration in the flow up to 172 ppm, and 329 ppm, respectively. Equation (1) is used to calculate the DRP concentration in ppm.

1 $$C_{DRP} = \frac{{Q_{DRP.} }}{{Q_{total} }} \times 1000$$

where *C*_*DRP*_ is the desired DRP concentration (ppm), *Q*_*DRP*_ is the flow rate of the DRP to be added (m^3^/s), and *Q*_*total*_ is the total liquid flow rate in the test section (m^3^/s).

**Figure 2:**
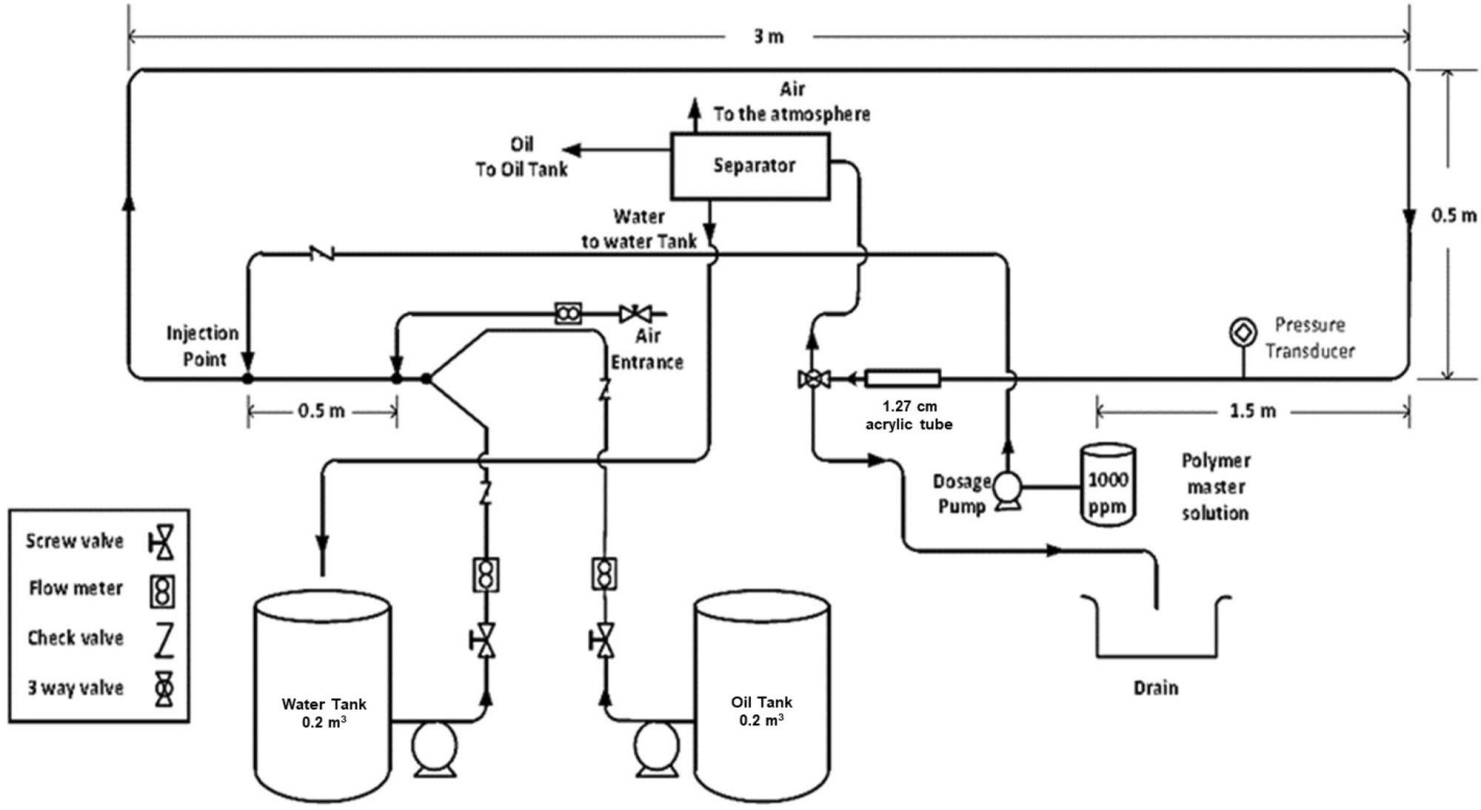
Schematics of the smaller diameter multi-phase flow facility.

The facility shown in Fig. 2 was used to investigate the effects of pipe diameter and operational conditions. The test section is made of 5 m long horizontal with an internal diameter of 10.16 mm stainless steel pipe, and the distance between the two pressure tabs is 1.5 m. This facility was designed to investigate the effect of DRP-WS on the flow behavior of the two-phase air–water mixture.

**Figure 3:**
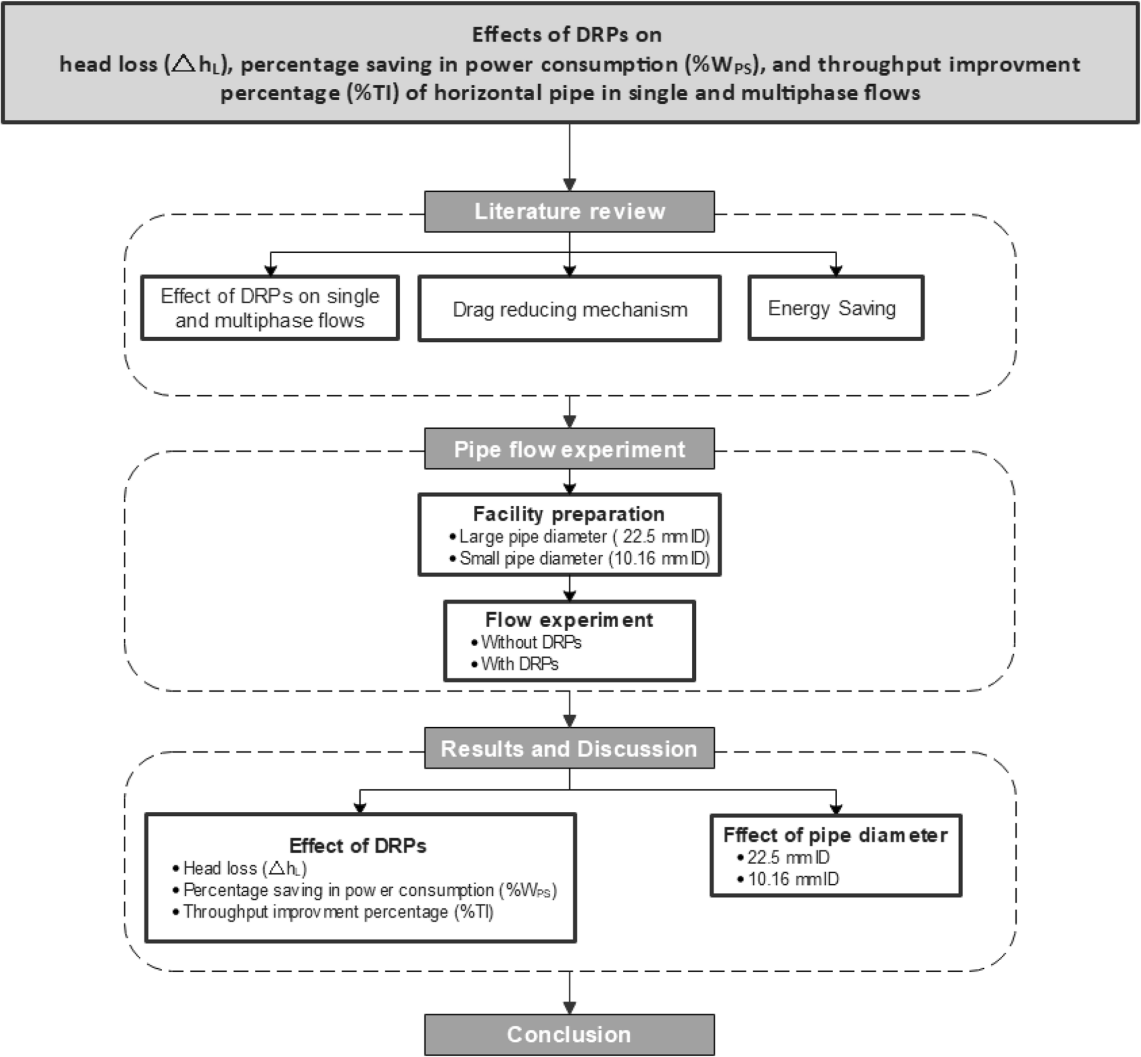
Flowchart of the current research approach.

Moreover, Fig. 3 summarizes the methodology of the research, starting with the effect of DRPs on the energy-efficiency metrics (head loss, percentage saving in energy consumption, and throughput improvement percentage) up to evaluate the use of DRPs as an additive to the fluid flow and draw qualitative and quantitative research outcomes.

### Energy analysis

#### Effect of DRPs on $\Delta {h}_{L}$

According to the Darcy-Weisbach equation, the head loss in single- and two-phase fully developed and turbulent pipe flow is proportional to the square of the liquid velocity. This equation is given as^45^:

2 $$\frac{{\Delta h_{L} }}{\Delta L} = \frac{\Delta P}{{\Delta L\gamma }} = f\frac{{U^{2} }}{2gD}$$

where *Δh*_*L*_ is the head loss (m), *ΔP* is the pressure drop (Pa), *γ* is the specific weight of fluid (N/m^3^), *f* refers to the fanning friction factor for smooth pipe, *U* is the average liquid velocity (m/s), *ΔL* is the distance between pressure tabs (m),* D* represents pipe diameter (m), and *g* is the gravity acceleration (m/s^2^).

For the three-phase flow, the head loss is calculated using Eq. (3):

3 $$\frac{{\Delta h_{L} }}{\Delta L} = \frac{\Delta P}{{\Delta L\gamma_{m} }} = f_{m} \frac{{U_{m}^{2} }}{2gD}$$

where *γ*_*m*_ refers to the liquid mixture specific weight (N/m^3^), *f*_*m*_ is the liquid mixture Fanning friction factor, and *U*_*m*_ is the liquid mixture velocity (m/s).

Liquid mixture Reynolds number and liquid mixture properties, such as density and viscosity, are given as the following:

4 $${Re}_{m}=\frac{{\rho }_{m}\times D\times {U}_{m}}{{\mu }_{m}}$$

5 $${\rho }_{m}={(\rho }_{Water}\times water cut)+({\rho }_{Oil}\times \left(1-water cut\right))$$

6 $${\mu }_{m}={(\mu }_{Water}\times water cut)+({\mu }_{Oil}\times \left(1-water cut\right))$$

7 $$water cut= \frac{{Q}_{Water}}{{Q}_{Water}+{Q}_{Oil}}$$

where ${\mathrm{Re}}_{\mathrm{m}}$: liquid mixture Reynolds number, ${\mu }_{m}$: Dynamic liquid mixture viscosity (N s/m^2^), ${\rho }_{Water}$: water density (kg/m^3^), ${\rho }_{Oil}$: oil density (kg/m^3^), ${Q}_{Water}$: volumetric water flow rate (m^3^/s), ${Q}_{Oil}$: volumetric oil flow rate (m^3^/s).

The percentage drag reduction (%DR) along the pipeline is given by:

8 $$\mathrm{\%}DR= \frac{\Delta {P}_{1}-\Delta {P}_{2}}{\Delta {P}_{1}}\times 100\mathrm{\%}$$

where *ΔP*_*1*_ : is the pressure drop measured without the DRP (Pa), *ΔP*_*2*_ : is the pressure drop measured with the DRP (Pa).

To evaluate the effect of adding DRPs on the head loss, Eqs. (2) or (3) were substituted in Eq. (8) as follows:

$$\mathrm{\%}DR=\frac{\gamma \Delta {h}_{L-1}-\gamma \Delta {h}_{L-2}}{\gamma \Delta {h}_{L-1}}\times 100\mathrm{\%} \;\;\;\;\; \text{or} \;\;\;\;\; \mathrm{\%}DR=\left[1-\frac{\Delta {h}_{L-2}}{\Delta {h}_{L-1}}\right]\times 100\mathrm{\%}$$

$$\frac{\mathrm{\%}DR}{100\mathrm{\%}}=\left[1-\frac{\Delta {h}_{L-2}}{\Delta {h}_{L-1}}\right] \;\;\;\;\;\;\;\;\;\;\;\; \text{or} \;\;\;\;\;\frac{\Delta {h}_{L-2}}{\Delta {h}_{L-1}}=\left[1-\frac{\mathrm{\%}DR}{100\mathrm{\%}}\right]$$

9 $$\Delta {h}_{L-2}=\Delta {h}_{L-1}\left[1-\frac{\mathrm{\%}DR}{100\mathrm{\%}}\right]$$

where *Δh*_*L-1*_: Head loss calculated in the absence of DRP per meter length (m/m), *Δh*_*L-2*_: Head loss calculated after adding the DRP per meter length (m/m).

#### Effect of DRPs on $\%{W}_{PS}$

The amount of saving in energy consumption due to adding DRP is an integral part of comprehensive energy analysis. This can be mathematically expressed as:

10 $${W}_{PS}={Q}_{m}{\gamma }_{m}\left(\Delta {h}_{L-1}-\Delta {h}_{L-2}\right)$$

where *W*_*PS*_ refers to the saving in power consumption per meter length (*W/m*) and *Q*_*m*_ is the mixture volumetric flow rate (m^3^/s), in which ${Q}_{m}={Q}_{Water}+{Q}_{Oil}$.

The percentage saving in energy consumption per length can be mathematically expressed as:

11 $$\%{W}_{PS}=\frac{{W}_{PS}}{W}\times 100\%$$

where *W* represents the power consumption per meter length (W/m), in which $W = Q_{m} \gamma_{m} \Delta h_{L - 1}$.

#### Effect of DRPs on $\%TI$

As a result of reducing the head loss, the pumpability will be enhanced likewise the flow rate or what is called the throughput. However, the throughput improvement is limited by the pressure the pipe can safely withstand. According to Lescarboura et al.^46^, the throughput improvement percentage (%TI) can be determined using Eq. (12):

12 $$\%TI=\left\{{\left[\frac{1}{1-\left(\%\frac{DR}{100}\right)}\right]}^{0.55}-1\right\}\times 100$$

## Results and discussions

In this section, the effects of injecting DRPs and flow combinations such as single-phase water flow, single-phase oil flow, two-phase air–water flow, two-phase air-oil flow, and three-phase air-oil–water flow on energy-efficiency indicators such as head loss, percentage saving in energy consumption, both per unit pipe length and throughput improvement percentage were addressed. Also, the outcomes of using different types of pipe diameters were discussed.

### Effect of flow combinations and DRPs

#### Head loss cutback by DRPs

This section specifically illustrates the varying effects of flow combinations and DRPs on decreasing head loss per unit pipe length Δh_L_/ΔL. Figures 5 and 6 investigate the effects of the experimental DRPs (DRP-WS and DRP-OS) on head loss per unit pipe length Δh_L_/ΔL in single-phase water flow, single-phase oil flow, two-phase air–water flow, two-phase air-oil flow, and three-phase air–oil–water flow. The test section comprises a horizontal pipe of 22.5 mm ID turbulent slug flow. Refer to Reynolds number presented in Tables S1 and S2, which are shown in the supplementary material. The head losses were calculated using Eqs. (2), (3), and (9). The results presented by these figures can be divided into two groups. Group I includes Figs. 5 and 6i, and Group II, Fig. 6ii. Groups I and II show the variation of Δh_L_/ΔL as the fluid flow rate Q_liquid_ and Q_air_ increase, respectively. Irrespective of single to multi-phase flows and the increase of Q_liquid_ and Q_air_, the influence of applying DRPs qualitatively remains the same. Moreover, visual observations revealed that both DRPs delayed the transition from low to high-frequency slug flow and the appearance of annular flow. They also reduced the pressure gradient dP/dL under all the experimental conditions, which in turn reduced the head loss per unit pipe length Δh_L_/ΔL, as a result, caused the drag-reducing. According to^33,41^, the presence of a small amount (in ppm scale) of high molecular weight polymers in the buffer region can inhibit the formation of turbulent bursts, as well as suppress the formation and dispersion of eddies in the turbulent region. These factors may contribute to the occurrence of drag reduction and, therefore, confirm the energy-saving capability of both DRPs in a highly turbulent flow.

**Figure 4:**
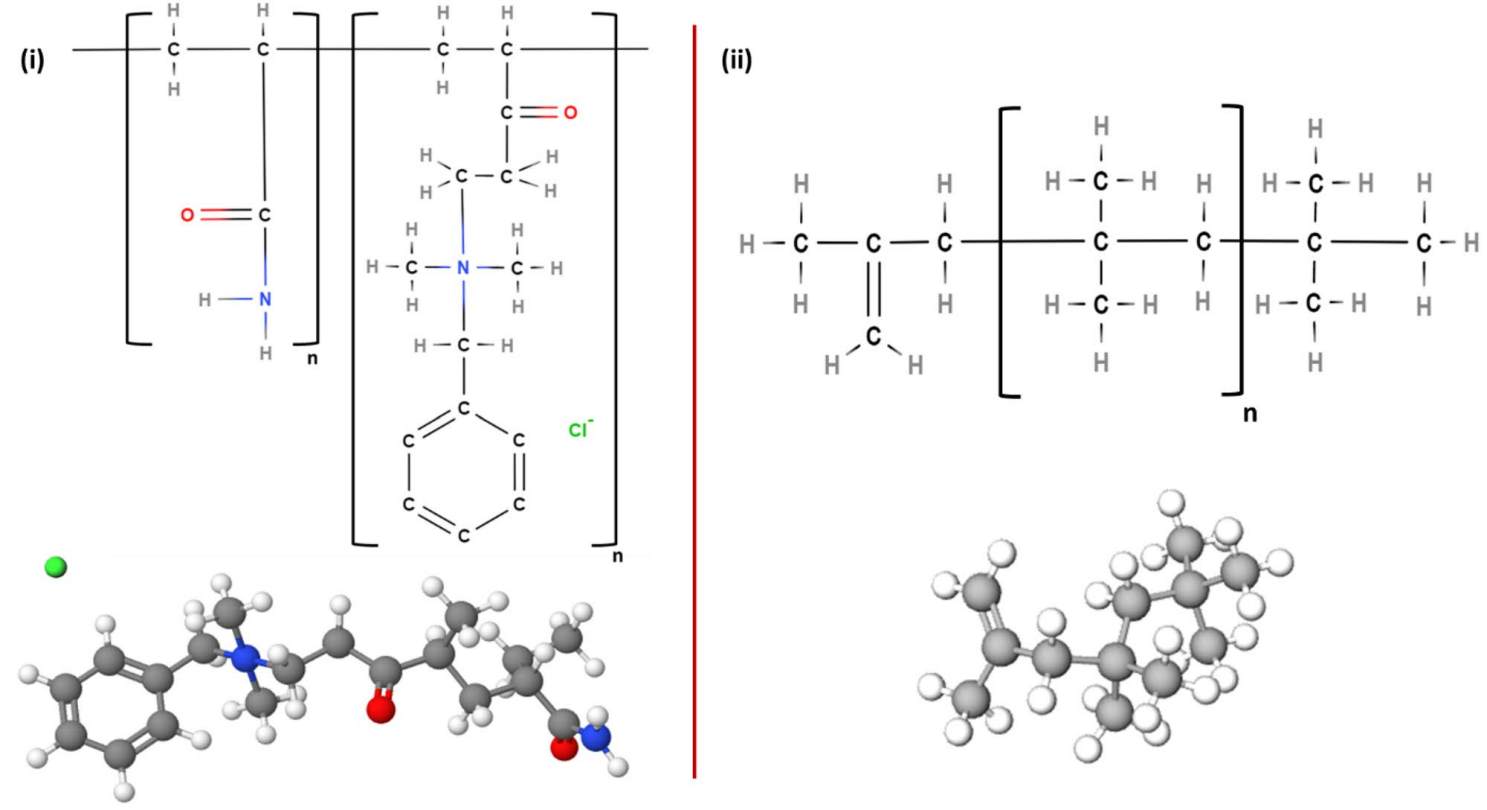
Chemical structures of (**i**) DRP-WS: copolymer of acrylamide and quaternized cationic monomer (polyacrylamide), and (**ii**) DRP-OS: polyisobutylene.

**Figure 5:**
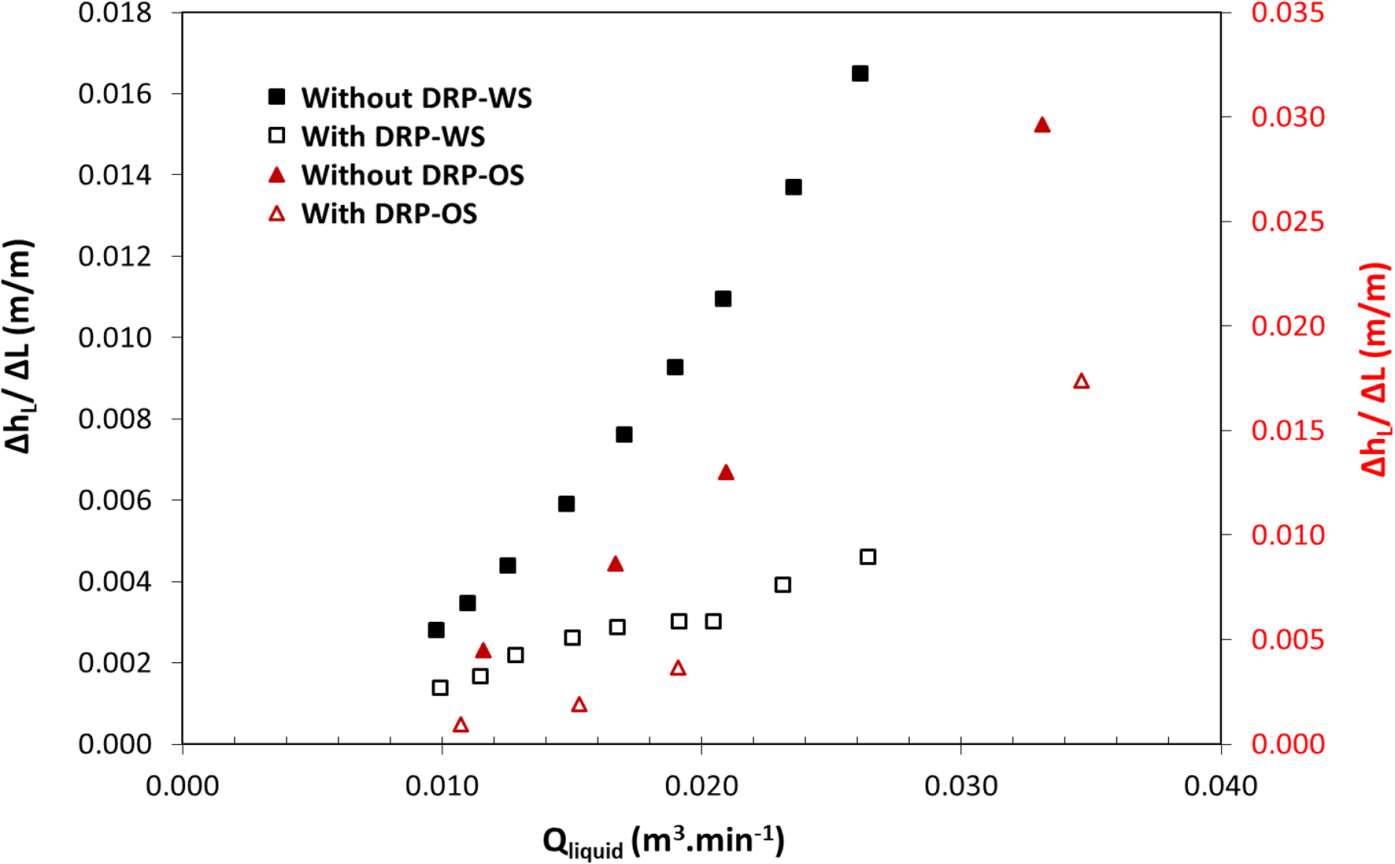
Head loss per meter length versus liquid flow rate in single-phase in horizontal pipe of 22.5 mm ID (**i**) black color represents water flow with and without DRP-WS at concentrations of 64-ppm up to 172-ppm (**ii**) red color represents oil flow with and without DRP-OS at concentrations of 101-ppm up to 329-ppm.

After establishing the above common finding that DRP decreases Δh_L_/ΔL, the single-phase flow experiments (Fig. 5) were considered for rating the energy-saving performance of both DRPs (DRP-WS and DRP-OS). Figure 5 shows a comparison in the performance of DRP-WS (at a concentration of 64 up to 172 ppm) over that of DRP-OS (at a concentration of 101 up to 329 ppm), where the concentration for both DRPs decreased with the increment of liquid flow rate, reveals that DRP-WS is more pronounced in reduction Δh_L_/ΔL than DRP-OS. This might be explained by the DRP-WS's ability to restrict the growth of turbulent eddies and the production of turbulent bursts since it has a higher molecular weight than the DRP-OS. This performance disparity can also be related to the fact that their structures differ, which in turn affects whether they have polar (DRP-WS) or nonpolar (DRP-OS) characteristics, as seen in Fig. 4^39^.

**Figure 6:**
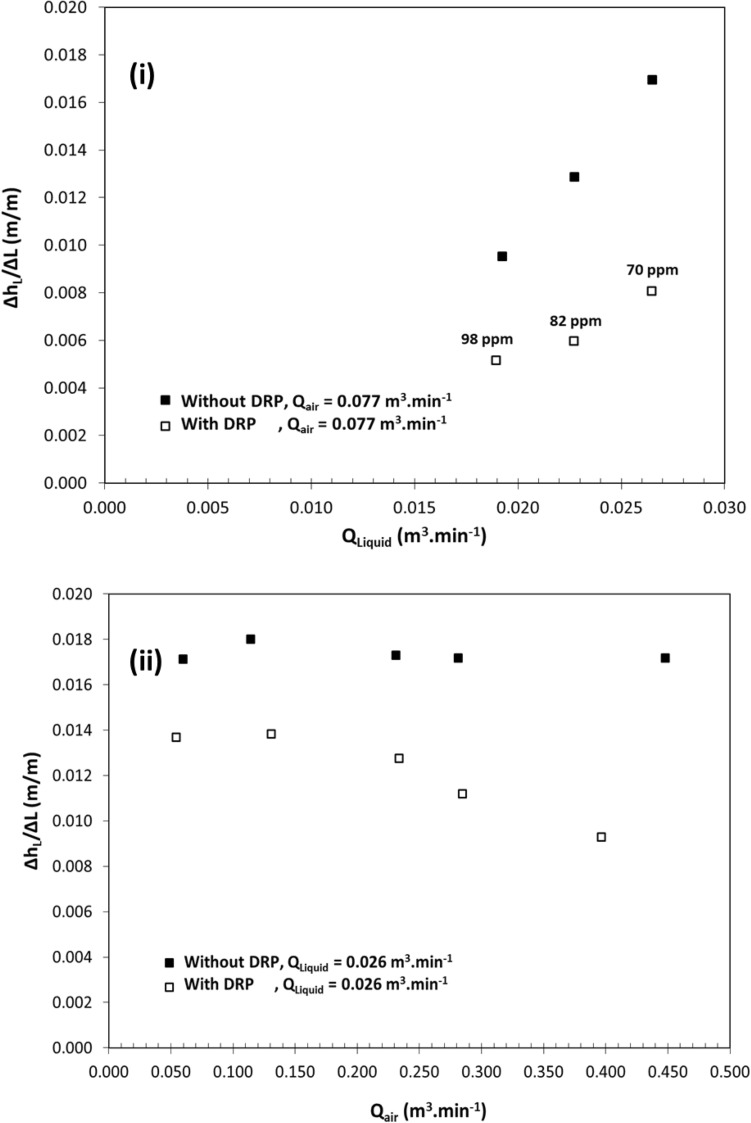
Head loss per meter length versus fluid flow rate in horizontal pipe of 22.5 mm ID for (**i**) two-phase air–water flow with and without DRP-WS at concentrations of 70-ppm up to 98-ppm, (**ii**) three-phase air-oil–water flow with and without DRP-WS at a concentration of 115-ppm.

Figure 6i shows the head loss reduction by DRP-WS at a concentrations of 70–98 ppm, which may be interpreted as the different level of interaction of DRP-WS with air–water turbulent bursts. This may be due to the different physical configurations of air–water two-phase flow in a pipe, knowing that the injection point of the DRP is always from the same location (side of the pipe). Figure 6ii shows a qualitatively matching conclusion which implies that DRP-WS reduced the head loss for each air flow rate applied. However, the observed effect on the head loss decreased as the air flow rate increased. In fact, the primary source of head loss reduction was the existence of DRP-WS in the water layer, which was governed by wall shear stress reduction and interfacial shear reduction between phases. This insightful result agrees with what Figs. 5 and 6i demonstrated.

#### Percentage saving in power consumption by DRPs

**Figure 7:**
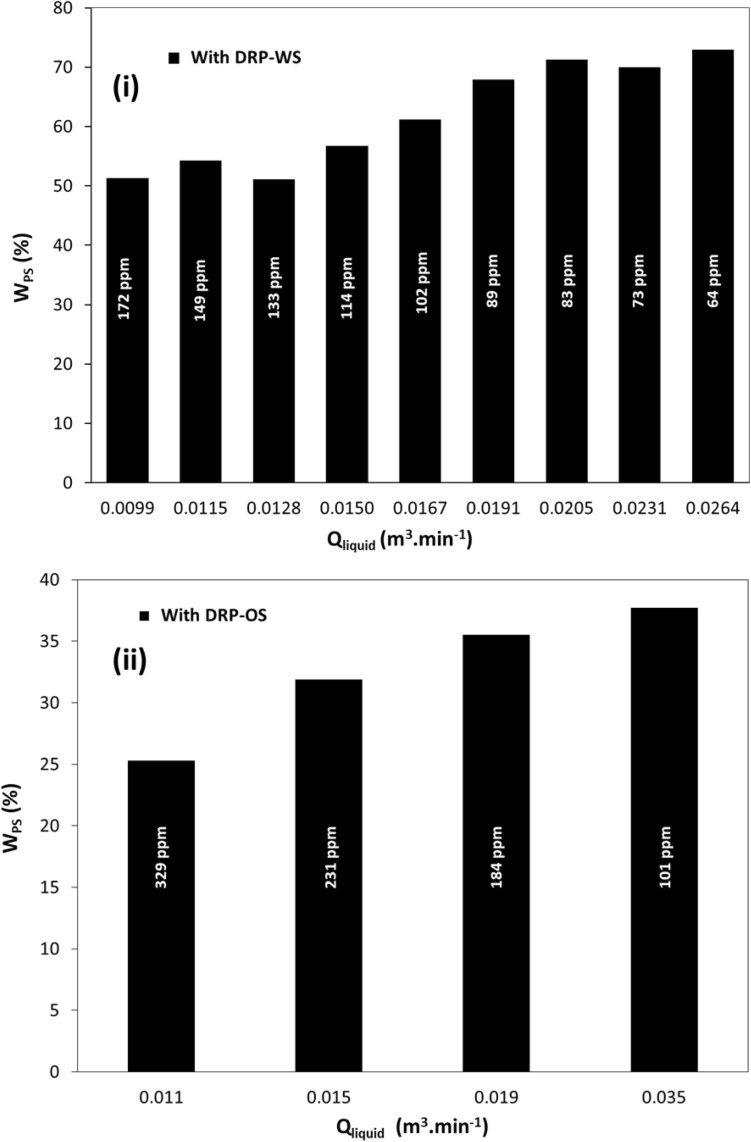
Percentage saving in power consumptions per meter length versus liquid flow rate in horizontal pipe of 22.5 mm ID for (**i**) DRP-WS (at concentrations of 64-ppm up to 172-ppm) with single-phase water flow, (**ii**) DRP-OS (at concentrations of 101-ppm up to 329-ppm) with single-phase oil flow.

**Figure 8:**
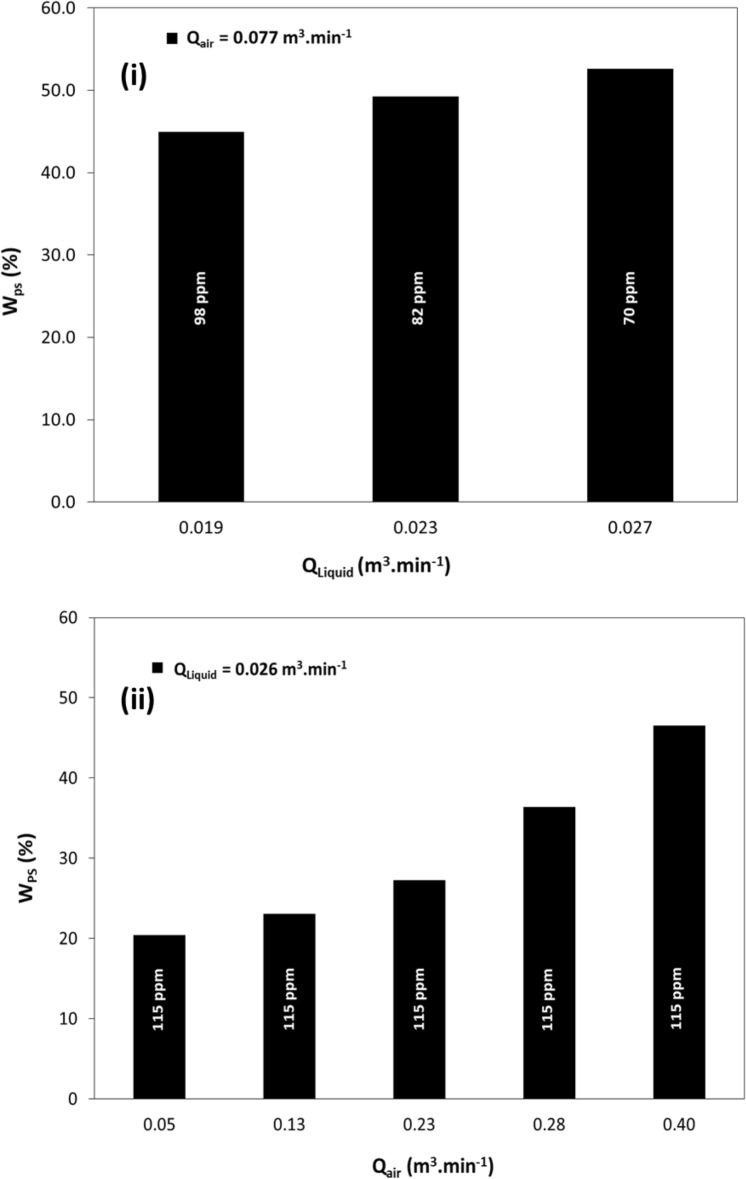
Percentage saving in power consumptions per meter length versus fluid flow rate in horizontal pipe of 22.5 mm ID for (**i**) DRP-WS at different concentrations with two-phase air–water flow, (**ii**) DRP-WS at the same concentration with three-phase air-oil–water flow.

This section discusses the impact of flow combinations and DRPs on reducing power consumption per unit length of the pipe, Wsp. The presence of DRPs results in aggregate energy savings, as indicated by Eq. (10), due to a reduction in head loss. This leads to a decrease in Δh_L_/ΔL and, subsequently, lower power consumption per unit length of the pipe. The effect of experimental DRPs on the percentage of power consumption saved per unit length of the pipe, %W_PS_, is shown in Figs. 7 and 8 as a function of fluid flow. The calculation of %W_PS_ is based on Eq. (11).

Figures 7 and 8 relate to increasing fluid flow rate, and the following general trend is noticed. For Fig. 7, %W_PS_ increased with the increase in Q_liquid,_ which leads to decrement in the DRPs concentration. Again, DRP-WS shows superior performance compared to DRP-OS. This can be interpreted as the DRP-OS's aggregate probably disintegrated due to the impingement of a very high liquid velocity on its continuum, which degraded the DRP-OS chain faster than the DRP-WS. This can be attributed to the difference in their structures.

The same trend is obtained as depicted in Fig. 7; %W_PS_ at a constant air flow rate (Fig. 7i) and/or liquid flow rate (Fig. 7ii) increased with the increase in Q_Liquid,_ which is lower than the decrement in %W_PS_ observed in Fig. 7i. This finding can be explained by considering that the air flow rate reduces the level of interaction between the DRP-WS molecules and the turbulent eddies of the water phase. Also, increasing the air flow rate increased the DRP degradation level.

#### Throughput improvement percentage (%TI) by DRPs

**Figure 9:**
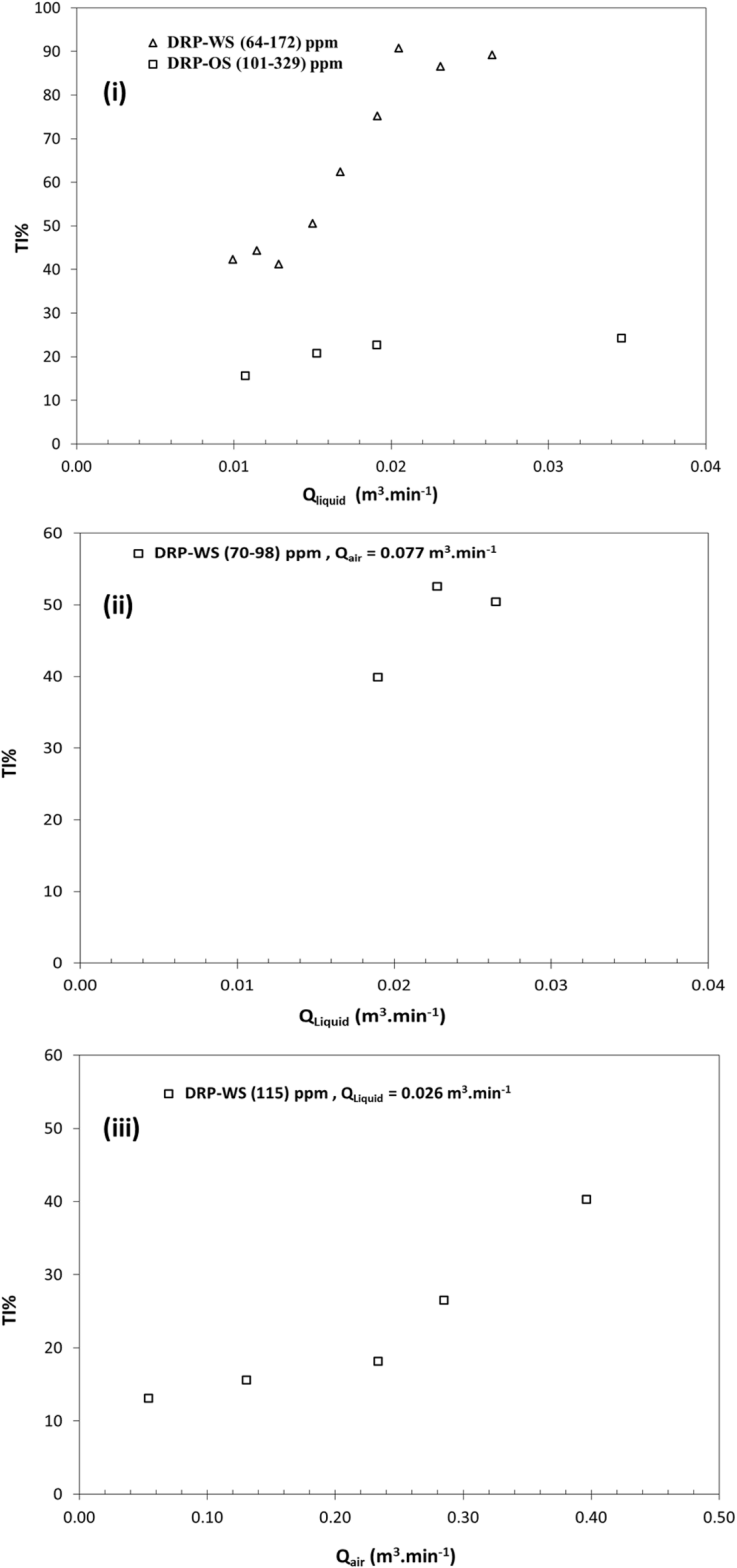
Throughput improvement percentage versus fluid flow rate in horizontal pipe of 22.5 mm ID for (**i**) single-phase water (open triangle) flow with DRP-WS and oil (open square) flow with DRP-OS, (**ii**) two-phase air–water flow with DRP-WS, (**iii**) three-phase air–oil–water flow with DRP-WS.

Another effective metric in the energy-efficiency enhancement is the implementation of Eq. (12). The throughput improvement percentage due to injecting DRPs falls between two limits; pump volumetric capacity and pipeline pressure. The maximum improvement in throughput for a pressure-limited line, which is the case here, is demonstrated in Fig. 9. The following results were observed:

- Figure 9i shows an increment of %TI up to 92% for DRP-WS at a concentration of 64 ppm. Meanwhile, at concentration of 101 ppm for DRP-OS, the maximum value of %TI of about 25%.
- Figure 9ii shows a high concentration of DRP-WS maintains a sort of stable performance of throughput increment as the liquid flow rate increases.
- Figure 9iii shows that the DRP-WS can significantly increase the throughput by up to 73%.

To sum up, Fig. 9i shows the pronouncing result of using DRP-WS over the DRP-OS. However, the trend of these figures coincided with the trends observed in previous sections, and the finding qualitatively conforms to that of single-phase, two-phase, and three-phase flows.

Based on the investigation conducted in the above energy saving metrics, it is increasingly apparent, as a result of using DRPs, a larger volume of fluid can be transported by using the same pump, or the same volume of fluid can be transported using a smaller pump. This means that neither situation saves energy.

### Effect of the pipe diameter

**Figure 10:**
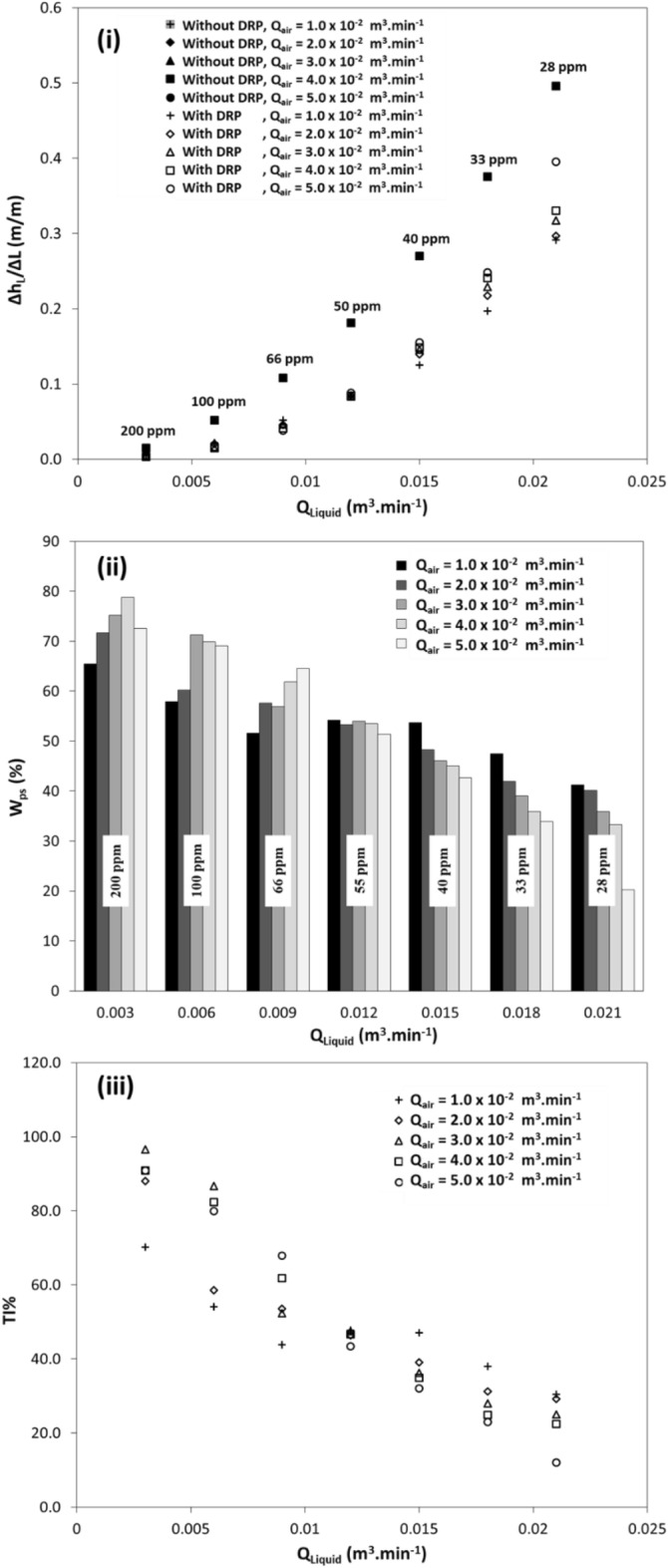
Two-phase air–water flow with and without DRP-WS at concentrations of 28-ppm up to 200-ppm in horizontal pipe of 10.16 mm ID versus (**i**) head loss per meter length versus liquid flow rate, (**ii**) percentage saving in power consumptions per meter length, (**iii**) throughput improvement percentage.

In this investigation, the effect of pipe diameter on head loss, percentage power saving, and throughput improvement percentage were evaluated considering the following as a case study:

i. DRP-WS;
ii. Two-phase air–water flow;
iii. 22.5 mm ID PVC pipe (Figs. 6i, 8i, and 9ii); and
iv. 10.16 mm ID stainless steel pipe (Fig. 10). More details are listed in Table S3.

Figure 5i relate to the head loss per unit pipe length Δh_L_/ΔL whereas Fig. 8i concern the percentage saving in power consumption per unit pipe length %W_PS_ and Fig. 9ii represents the throughput improvement percentage %TI in the above 22.5 mm PVC pipe. Figure 10i corresponds to Fig. 6i, however, in the 10.16 mm ID stainless steel pipe. Figure 10ii is the analog of Fig. 8. Consequently, Fig. 10iii is related to Fig. 9ii.

The comparison of results represented by the above figures shows that the head loss Δh_L_/ΔL drastically increased in the smaller pipe. The effect on the percentage saving in power consumption %W_PS_ and the throughput improvement percentage %TI are opposite. %W_PS_ and %TI in the smaller pipe diameter were found to be significantly comparable with low DRP-WS concentrations in the larger pipe diameter (Figs. 8i and 9ii). This can be attributed to the significant level of turbulence, and the frictional pressure gradient in the smaller pipe diameter compared to the larger diameter. These results are in good agreement with the findings of^31^. According to the elastic sub-layer model proposed by Virk^47^, as the concentration of additives increases, the elastic sub-layer expands, and the friction factor decreases. Savins^48^ was the first to describe the "diameter effect," which suggests that the influence of drag-reducing polymers on boundary layer flow is more substantial in smaller pipes because the boundary layer makes up a larger proportion of the total flow in these pipes. As the pipe diameter increases, the effect of the polymers on the flow decreases.

## Conclusions

Improving energy efficiency in crucial industrial applications, drag-reducing polymers (DRPs) are a much better choice because they do not require any extra infrastructure. So, utilizing single-phase water flow, single-phase oil flow, two-phase air–water flow, two-phase air-oil flow, and three-phase air–oil–water flow, this study assesses the impact of two DRPs—one nonpolar oil-soluble and polar (DRP-OS) and the other water-soluble and polar (DRP-WS)—on energy efficiency. The pipe comprises a horizontal pipe of 22.5 mm ID. The energy analysis is performed by investigating the head loss, saving in energy consumption (both per unit pipe length), and throughput improvement percentage.

In spite of switching from single to multi-phase flows as well as an increase in liquid and air flow rates, DRPs reduce head loss and boost energy savings. The total observation therefore reveals that the injection of DRPs into single and multi-phase flows tends to boost the throughputs up to 93% in certain cases. Notwithstanding, based on the conducted experiments, DRP-WS with concentrations ranging from 60 to 115 ppm is more energy efficient than DRP-OS.

Equivalent experiments conducted using the DRP-WS and the two-phase air–water flow in the 10.16 mm ID stainless steel pipe show that the head loss drastically increases in the smaller pipe. However, here the percentage saving in power consumption and throughput improvement percentage is significantly compared with that found in the larger 22.5 mm diameter pipe.

Based on the conclusions of this study, future research should focus on the following directives:

- Further investigation of the energy-saving of DRP-WS in various flow systems and determining the optimal concentration and molecular weight of DRPs for maximizing energy efficiency in different flow systems.
- More research is needed into the ways that DRPs increase energy efficiency under various flow system conditions.
- Evaluating the potential of DRPs to reduce energy consumption, improve energy efficiency, reduce friction factor, and reduce the heat transfer in industrial applications that involves laminar, transition, and turbulent flow regimes.

## Supplementary Information

Supplementary Tables.

## Data Availability

Electronic supplementary materials, including data and other relevant information, will be available to view and download upon publication of the manuscript. For support please contact Ihab H. Alsurakji isurakji@najah.edu.

## List of symbols

*DRP-WS*: Drag reducing polymer-water soluble
*DRP-OS*: Drag reducing polymer-oil soluble
*Δh*_*L*_: Head loss [m]
*ΔP*: Pressure drop [Pa]
*f*: Fanning friction factor
*L*: Pipe length [m]
*U*: Average velocity [m/s]
*D*: Pipe diameter [m]
*g*: Gravitational acceleration [m/s^2^]
Re: Reynolds number
*V*_*S*_: Superficial velocity [m/s]
*%DR*: Percentage drag reduction
*W*: Energy consumption [W/m]
*Q*: Volumetric flow rate [m^3^/s] or [m^3^/min]
*C*: Concentration [ppm

## Greek symbols

*γ*: Specific weight [N/m^3^]
*µ*: Dynamic viscosity [N s/m^2^

## Subscripts

*m*: Mixture
*1*: Without DRP
*2*: With DRP
*W*: Water
*O*: Oil
*PS*: Power saving
*DRP*: Drag reducing polymer
