# Supplementary material for: Experimental insights into energy savings and future directions of drag reducing polymers in multiphase flow pipelines
Source: Sci Rep. 2023 Jun 30;13:10619. doi: 10.1038/s41598-023-37543-w (PMC10313646; doi:10.1038/s41598-023-37543-w)
Supplement: Supplementary file 1 — Supplementary Tables. [file 41598_2023_37543_MOESM1_ESM.docx]

Experimental Insights into Energy Savings and Future Directions of Drag Reducing Polymers in Multiphase Flow Pipelines

**Ihab H. Alsurakji^1,*^,** **A. Al-Sarkhi^2^,** **Amjad El-Qanni^3^,** **Ayman Mukhaimar^4^**

^1^ An-Najah National University, Department of Mechanical Engineering, Nablus 7, Palestine

^2^ King Fahd University of Petroleum & Minerals, Department of Mechanical Engineering, Dhahran 31261, KSA

^3^ An-Najah National University, Department of Chemical Engineering, Nablus 7, Palestine

^4^ RMIT University, School of Computing Technologies, Melbourne, Australia

**^*^***Corresponding authors*: [*isurakji@najah.edu*](mailto:isurakji@najah.edu)*; Tel.: +970595814481;*

**Supplementary Materials**

**Table S1 Experimental test matrix for DRP-WS (22.5 mm ID).**

|  | **DRP-WS** | | | | | | | | | | | |
| --- | --- | --- | --- | --- | --- | --- | --- | --- | --- | --- | --- | --- |
| **Types** | V_SW_  (m.sec^-1^) | V_SO_  (m.sec^-1^) | V_SG_  (m.sec^-1^) | Q_pol._  (m^3^.min^-1^) | Q_Liquid_  (m^3^.min^-1^) | F.P. without DRP | F.P. with DRP | Max.  %DR | Re_SW_ | Re_SO_ | Re_SG_ | C  ppm |
| **Single- phase** | 0.344 | -- | -- | 0.0017 | 0.00991 | SP | SP | 50.6 | 9,479 | -- | -- | 172 |
|  | 0.408 | -- | -- | 0.0017 | 0.01145 | SP | SP | 52 | 10,951 | -- | -- | 149 |
|  | 0.466 | -- | -- | 0.0017 | 0.01284 | SP | SP | 50 | 12,271 | -- | -- | 133 |
|  | 0.557 | -- | -- | 0.0017 | 0.01500 | SP | SP | 56 | 14,343 | -- | -- | 114 |
|  | 0.630 | -- | -- | 0.0017 | 0.01674 | SP | SP | 62 | 16,008 | -- | -- | 102 |
|  | 0.730 | -- | -- | 0.0017 | 0.01911 | SP | SP | 67.4 | 18,274 | -- | -- | 89 |
|  | 0.801 | -- | -- | 0.0017 | 0.02046 | SP | SP | 72.5 | 19,563 | -- | -- | 83 |
|  | 0.858 | -- | -- | 0.0017 | 0.02313 | SP | SP | 71.3 | 22,112 | -- | -- | 73 |
|  | 1.036 | -- | -- | 0.0017 | 0.02642 | SP | SP | 72 | 25,261 | -- | -- | 64 |
| **Two-Phase** | 0.74 | -- | 3.31 | 0.0–0.0035 | 0.019 | S | SLF | 46 | 16,869 | -- | 4,965 | 98 |
|  | 0.95 | -- | 3.31 | 0.0–0.0035 | 0.0227 | S | SLF | 54 | 21,657 | -- | 4,965 | 82 |
|  | 1.10 | -- | 3.85 | 0.0–0.0035 | 0.0265 | S | SLF | 52 | 25,076 | -- | 5,775 | 70 |
| **Three-Phase** | 0.666 | 0.316 | 2.27 | 0.003 | 0.026 | S | SLF | 20 | 11,816 | 5,606 | 3,405 | 115 |
|  | 0.666 | 0.316 | 5.5 | 0.003 | 0.026 | SHF | SLF | 23 | 11,816 | 5,606 | 8,250 | 115 |
|  | 0.666 | 0.316 | 9.8 | 0.003 | 0.026 | SHF | SLF | 26 | 11,816 | 5,606 | 14,700 | 115 |
|  | 0.666 | 0.316 | 12.0 | 0.003 | 0.026 | SHF | SLF | 35 | 11,816 | 5,606 | 18,000 | 115 |
|  | 0.666 | 0.316 | 16.6 | 0.003 | 0.026 | A | SHF | 45 | 11,816 | 5,606 | 24,900 | 115 |

**SP:** Single-phase flow; **S:** Slug flow; **SLF:** Slug low-frequency flow; **SHF:** Slug high-frequency flow; **A:** Annular flow

**Table S2 Experimental test matrix for DRP-OS (22.5 mm ID).**

|  | **DRP-OS** | | | | | | | | | | | |
| --- | --- | --- | --- | --- | --- | --- | --- | --- | --- | --- | --- | --- |
| **Types** | V_SW_  (m.sec^-1^) | V_SO_  (m.sec^-1^) | V_SG_  (m.sec^-1^) | Q_pol._  (m^3^.min^-1^) | Q_Liquid_  (m^3^.min^-1^) | F.P. without DRP | F.P. with DRP | Max.  %DR | Re_SW_ | Re_SO_ | Re_SG_ | C  ppm |
| **Single- Phase** | -- | 1.35 | -- | 0.0024 | 0.035 | SP | SP | 41.4 | -- | 13,934 | -- | 329 |
|  | -- | 0.92 | -- | 0.0024 | 0.0245 | SP | SP | 71.7 | -- | 9,496 | -- | 231 |
|  | -- | 0.54 | -- | 0.0024 | 0.0153 | SP | SP | 77.9 | -- | 5,574 | -- | 184 |
|  | -- | 0.35 | -- | 0.0024 | 0.0107 | SP | SP | 79.3 | -- | 3,612 | -- | 101 |

**SP:** Single-phase flow

**Table S3 Experimental test matrix and observed percentage drag reduction for two-phase air-water with**

**DRP-WS (10.16 mm ID).**

|  | **DRP-WS** | | | | | | | |
| --- | --- | --- | --- | --- | --- | --- | --- | --- |
| Type | V_SW_  (m.sec^-1^) | V_SG_  (m.sec^-1^) | Q_air_  (m^3^.min^-1^) | Q_water_  (m^3^.min^-1^) | DR% | Re_SW_ | Re_SG_ | C  ppm |
| Two-Phase | 0.617 | 2.056 | 1.67E-04 | 0.003 | 65 | 7032 | 1445 | 200 |
|  | 0.617 | 4.112 | 3.33E-04 | 0.003 | 72 | 7032 | 2890 | 200 |
|  | 0.617 | 6.167 | 5.00E-04 | 0.003 | 75 | 7032 | 4335 | 200 |
|  | 0.617 | 8.223 | 6.67E-04 | 0.003 | 79 | 7032 | 5780 | 200 |
|  | 0.617 | 10.279 | 8.33E-04 | 0.003 | 73 | 7032 | 7226 | 200 |
|  | 1.233 | 2.056 | 1.67E-04 | 0.006 | 58 | 14065 | 1445 | 100 |
|  | 1.233 | 4.112 | 3.33E-04 | 0.006 | 60 | 14065 | 2890 | 100 |
|  | 1.233 | 6.167 | 5.00E-04 | 0.006 | 71 | 14065 | 4335 | 100 |
|  | 1.233 | 8.223 | 6.67E-04 | 0.006 | 70 | 14065 | 5780 | 100 |
|  | 1.233 | 10.279 | 8.33E-04 | 0.006 | 69 | 14065 | 7226 | 100 |
|  | 1.850 | 2.056 | 1.67E-04 | 0.009 | 52 | 21097 | 1445 | 66 |
|  | 1.850 | 4.112 | 3.33E-04 | 0.009 | 58 | 21097 | 2890 | 66 |
|  | 1.850 | 6.167 | 5.00E-04 | 0.009 | 57 | 21097 | 4335 | 66 |
|  | 1.850 | 8.223 | 6.67E-04 | 0.009 | 62 | 21097 | 5780 | 66 |
|  | 1.850 | 10.279 | 8.33E-04 | 0.009 | 65 | 21097 | 7226 | 66 |
|  | 2.467 | 2.056 | 1.67E-04 | 0.012 | 54 | 28130 | 1445 | 50 |
|  | 2.467 | 4.112 | 3.33E-04 | 0.012 | 53 | 28130 | 2890 | 50 |
|  | 2.467 | 6.167 | 5.00E-04 | 0.012 | 54 | 28130 | 4335 | 50 |
|  | 2.467 | 8.223 | 6.67E-04 | 0.012 | 54 | 28130 | 5780 | 50 |
|  | 2.467 | 10.279 | 8.33E-04 | 0.012 | 51 | 28130 | 7226 | 50 |
|  | 3.084 | 2.056 | 1.67E-04 | 0.015 | 54 | 35162 | 1445 | 40 |
|  | 3.084 | 4.112 | 3.33E-04 | 0.015 | 48 | 35162 | 2890 | 40 |
|  | 3.084 | 6.167 | 5.00E-04 | 0.015 | 46 | 35162 | 4335 | 40 |
|  | 3.084 | 8.223 | 6.67E-04 | 0.015 | 45 | 35162 | 5780 | 40 |
|  | 3.084 | 10.279 | 8.33E-04 | 0.015 | 43 | 35162 | 7226 | 40 |
|  | 3.700 | 2.056 | 1.67E-04 | 0.018 | 48 | 42195 | 1445 | 33 |
|  | 3.700 | 4.112 | 3.33E-04 | 0.018 | 42 | 42195 | 2890 | 33 |
|  | 3.700 | 6.167 | 5.00E-04 | 0.018 | 39 | 42195 | 4335 | 33 |
|  | 3.700 | 8.223 | 6.67E-04 | 0.018 | 36 | 42195 | 5780 | 33 |
|  | 3.700 | 10.279 | 8.33E-04 | 0.018 | 34 | 42195 | 7226 | 33 |
|  | 4.317 | 2.056 | 1.67E-04 | 0.021 | 41 | 49227 | 1445 | 28 |
|  | 4.317 | 4.112 | 3.33E-04 | 0.021 | 40 | 49227 | 2890 | 28 |
|  | 4.317 | 6.167 | 5.00E-04 | 0.021 | 36 | 49227 | 4335 | 28 |
|  | 4.317 | 8.223 | 6.67E-04 | 0.021 | 33 | 49227 | 5780 | 28 |
|  | 4.317 | 10.279 | 8.33E-04 | 0.021 | 20 | 49227 | 7226 | 28 |
